# Prevalence and prognostic impact of retropharyngeal lymph nodes metastases in oropharyngeal squamous cell carcinoma: Meta‐analysis of published literature

**DOI:** 10.1002/hed.27166

**Published:** 2022-08-01

**Authors:** Giancarlo Tirelli, Nicoletta Gardenal, Enrico Zanelli, Daniele Borsetto, Veronica Phillips, Alberto Vito Marcuzzo, Jonathan Fussey, Jerry Polesel, Paolo Boscolo‐Rizzo

**Affiliations:** ^1^ Department of Medical, Surgical, and Health Sciences, Section of Otolaryngology University of Trieste Trieste Italy; ^2^ Department of ENT Addenbrooke's Hospital, Cambridge University Hospitals NHS Foundation Trust Cambridge UK; ^3^ University of Cambridge Medical Library Cambridge UK; ^4^ Department of ENT/Head and Neck Surgery Queen Elizabeth University Hospital Birmingham Birmingham UK; ^5^ Unit of Cancer Epidemiology Centro di Riferimento Oncologico di Aviano (CRO) IRCCS Aviano Italy

**Keywords:** HPV, human papillomavirus, oropharyngeal cancer, retropharyngeal lymph nodes, retropharyngeal metastasis

## Abstract

**Background:**

This systematic review and meta‐analysis aims to estimate the prevalence and prognostic impact of retropharyngeal lymph node metastases (RLNMs) in oropharyngeal squamous cell carcinoma (OPSCC).

**Methods:**

This meta‐analysis was conducted according to PRISMA guidelines. Inclusion criteria: studies with more than 20 patients reporting the prevalence or prognostic impact of RLNMs in OPSCC. Whenever available, data on HPV status and subsites were extracted.

**Results:**

Twenty‐two articles were included. The overall prevalence of RLNMs in OPSCC was 13%, with no significant differences depending on HPV status. The highest prevalence was observed for posterior pharyngeal wall SCC (24%), followed by soft palate (17%), palatine tonsil (15%), and base of tongue (8%). RLNMs were associated with a significantly higher risk of death (HR:2.54;IC95%1.89–3.41) and progression (HR:2.44;IC95%1.80–3.30).

**Conclusions:**

The prevalence of RLNMs in OPSCC was 13%, being higher in tumors of the posterior pharyngeal wall. RLNMs were associated with unfavorable outcomes.

## 1 INTRODUCTION

The management of retropharyngeal lymph nodes (RLNs) in head and neck squamous carcinoma (HNSCC) is still an open issue.^1^ Anatomically, RLNs are located within a thin fat pad in the retropharyngeal space which is delimited anteriorly by the pharynx and its buccopharyngeal fascia and posteriorly by the alar fascia which itself forms the anterior aspect of the danger space and the prevertebral plane. RLNs are classified in medial and lateral groups.^2^

Robust data regarding the prevalence of RLN metastases (RLNMs) are lacking given that these lymph node chains are relatively inaccessible to clinical examination and fine needle aspiration biopsy, and they are not routinely removed in neck dissection.^3^ Therefore, there are insufficient clinicopathological correlations, such as size and shape of the nodes, to be able to define radiological criteria suggestive of their metastatic involvement.^4^, ^5^ Consequently, the prevalence of RLNMs varies widely in the literature. Based on a few histological and radiological studies, rates of RLNMs in HNSCC ranges from 9% to 50%,^1^ with nasopharyngeal carcinoma (NPC) exhibiting the greatest propensity to metastasize to the RLNs (29–89%).^6^ Within non‐nasopharyngeal HNSCCs, oropharyngeal squamous cell carcinomas (OPSCCs) have been reported to have the highest rate of RLNMs, although there is a wide range of prevalence across studies, ranging from 6% to 23%.^1^ Given the uncertainty regarding the real rate of RLNMs, its prognostic impact is controversial, especially considering the heterogeneous interpretations of results predominantly from small case series.^7^, ^8^, ^9^, ^10^ Even more inconsistent results have been reported when considering the potential impact of human papillomavirus (HPV) status and the different oropharyngeal subsites on the rate of metastasis to the RLNs.^11^, ^12^

This systematic review and meta‐analysis aims to summarize the current evidence on the prevalence of RLN metastasis in OPSCCs and on its prognostic relevance, with a focus on HPV status and different oropharyngeal subsites.

## 2 METHODS

### 2.1 Outcome measures

The primary outcome measure was the prevalence of metastases in RLNs in OPSCC, defined as the number of patients with metastatic involvement of the RLN/total number of patients. Pooled prevalence was estimated separately depending on the diagnostic tool used to assess the RLN metastatic involvement, that is, histopathological or imaging evaluation. The rate of RLNMs stratified by HPV status (as detected by p16 immunohistochemistry, HPV‐DNA in situ hybridization or PCR) and by OPSCC subsites (tonsil, base of the tongue, lateral and posterior pharyngeal wall, and soft palate) were also explored. The secondary outcome of this meta‐analysis was the prognostic impact of RLNMs for the following outcomes, (a) overall survival (OS), defined as the time from diagnosis or initiation of treatment to patient death, irrespective of cause; (b) disease free survival (DFS), defined as the time from diagnosis or initiation of treatment until tumor recurrence/progression or any‐cause death; (c) loco‐regional control (LRC), defined as the time from diagnosis or initiation of treatment to the first locoregional event; and (d) distant metastasis (DM), defined as the time from diagnosis or initiation of treatment to the first distant event.

### 2.2 Search strategy

**FIGURE 1:**
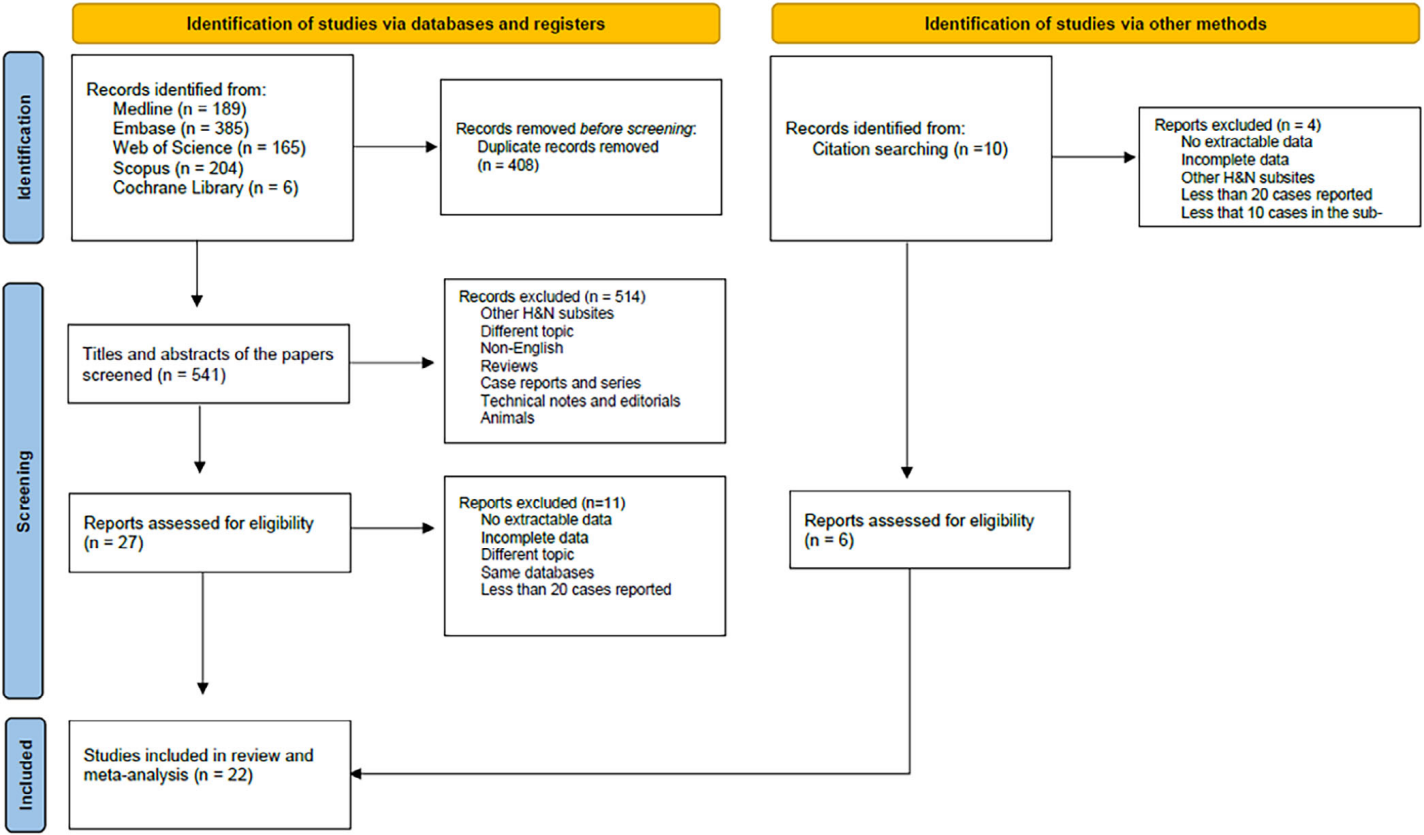
PRISMA flow diagram [Color figure can be viewed at wileyonlinelibrary.com]

This systematic review and meta‐analysis was conducted following the preferred reporting items for systematic reviews and meta‐analysis (PRISMA) checklist.^13^ The databases Medline (via Ovid), Embase (via Ovid), Cochrane Library, Web of Science (core collection), and Scopus were searched from inception to May 2021. The search terms used are reported below. Prior to searching the databases, the search terms were peer reviewed by three authors (PBR, DB, and EZ) to ensure they conformed to PRESS guidelines.^14^ “Head and Neck Neoplasms” OR “Esophageal Neoplasm” OR “Facial Neoplasm” OR “Mouth Neoplasm” OR “Otorhinolaryngologic Neoplasm” OR “Tracheal Neoplasm” OR “Head and Neck neoplasm” OR “Carcinoma” OR “Squamous Cell” OR “Mouth Neoplasms” OR “Oral Cavity Neoplasm” OR “Oropharyngeal Neoplasms” OR “Oropharyngeal Neoplasm” OR “Hypopharyngeal Neoplasms” OR “Hypopharyngeal Neoplasm” OR “Head and Neck Cancer” OR “Esophageal Cancer” OR Mouth Cancer” OR “Otorhinolaryngologic cancer” OR “Parathyroid Cancer” OR “Thyroid Cancer” OR “Trachea Cancer” “Squamous Cell Carcinoma” OR “Oropharyngeal Cancer” OR “Hypopharyngeal Cancer” AND “Retropharyngeal Node.” The reference list of articles included in this review were also manually searched to minimize the risk of data loss. Two authors (DB and EZ) independently screened all titles and abstracts identified by the search and then evaluated the full text of the reports that respected the inclusion criteria. A third author (PBR) settled any disagreement between reviewers. A flow diagram illustrating all the steps that led to the selection of the reports eligible for meta‐analysis is shown in Figure 1.

### 2.3 Inclusion criteria

Studies were included in the analysis if they met the following criteria: (1) studies reporting the prevalence of RLNMs, based on imaging, that is, computed tomography (CT), magnetic resonance imaging (MRI), or positron emission tomography/CT (PET/CT), or pathological examination and/or the prognostic role of RLNMs in OPSCC; (2) studies in which RLN involvement was assessed radiologically or by histopathological examination; (3) studies including at least 20 patients with OPSCC; and (4) studies reporting sufficient data for statistical analysis. Authors were contacted by email if a study met the inclusion criteria, but data were not immediately extractable. Non‐English language studies were excluded. Studies containing aggregated data or duplicated data from previously published work were excluded, as were review articles, case reports, editorials, and letters. Two authors (JP, PBR) independently assessed the quality of the included studies with the Newcastle‐Ottawa Scale.^15^ Low‐quality articles (Newcastle–Ottawa Scale [NOS] score <6) were also excluded.

### 2.4 Data extraction and statistical analysis

The number of total cases and of positive RLNs were extracted from each study. When available, data were extracted according to HPV status and cancer subsite. To provide a summary estimate of the association between RLN‐positivity and oncological outcomes, the hazard ratio (HR) was extracted, when reported. The standard error of the log hazard ratio (HR) was derived from the log confidence intervals (CIs). Summary estimates of proportion or hazard ratio (sHR), with corresponding 95% CI, were calculated according to random‐effects models of DerSimonian and Laird,^16^ which incorporates both within‐ and between‐study variability, as a weighted average giving each study a weight proportional to its precision. Analyses by strata according to HPV status and cancer subsite were conducted including studies with ≥10 cases in the stratum under investigation. Statistical heterogeneity among studies was evaluated using the *I* ^2^ and τ^2^ statistics.^16^ Influence analysis was performed when the summary estimate was estimated from five or more studies: the summary estimate was calculated by omitting one study at a time. Publication bias was assessed through a funnel plot.^17^ The results of the meta‐analysis were presented graphically using forest plots, plotting the estimates from individual papers, summary estimate, and 95% CI. Statistical significance was claimed for *p* < 0.05.

## 3 RESULTS

### 3.1 Search results and study characteristics

We identified a total of 949 potentially relevant articles with our initial search strategy. After screening the titles and abstracts of these articles, we excluded 922 studies because they were duplicated, or they did not meet the inclusion criteria (Figure 1). After reading 27 potentially eligible articles in detail, we determined that 16 studies met our inclusion criteria: the study by Bussels et al.^18^ was excluded because it reported the same cohort as of Dirix et al.^7^ Ten additional studies,^4^, ^11^, ^19^, ^20^, ^21^, ^22^, ^23^, ^24^, ^25^, ^26^ of which six^11^, ^19^, ^20^, ^21^, ^22^, ^23^ were eligible for meta‐analysis, were identified through checking the reference lists of initial 27 eligible articles. Overall, 22 articles^7^, ^8^, ^9^, ^10^, ^11^, ^12^, ^19^, ^20^, ^21^, ^22^, ^23^, ^27^, ^28^, ^29^, ^30^, ^31^, ^32^, ^33^, ^34^, ^35^, ^36^, ^37^ were included in the final analysis including 5027 patients with OPSCC. Among studies reporting the involved subsites of the oropharynx: 2242 subjects (52.1%) had palatine tonsil SCC, 1689 (39.3%) had SCC of the base of the tongue (BOT), 187 (4.3%) and 150 (3.4%) had posterior pharyngeal wall (PPW) and soft palate SCC, respectively. Thirteen authors^10^, ^11^, ^12^, ^19^, ^20^, ^25^, ^27^, ^37^, ^38^, ^39^, ^40^, ^41^, ^42^ were contacted in order to gather useful data to include in the metanalysis but only one provided the requested information.^11^ The characteristics of the included studies are presented in Table 1.

**TABLE 1:** Description of included studies

Study	Study type	No. of patients	Cohort details	RLN diagnosis	Radiology criteria	Treatment
Rosen, 2021^27^	R	266	OPSCC	CT‐PET/CT	LN >8 mm, SUV > 2.5	RT/RTCT
Billfalk, 2019^11^	R	280	cT1‐T2 N1 OPSCC HPV+	CT‐MRI	LN >8 mm	RT/ CTRT
Iyizoba‐Ebozue, 2020^28^	R	402	OPSCC	CT‐MRI‐PET/CT	LN short axis ≥5 mm, necrosis and/or abnormal uptake on PET‐CT	RT/RTCT
Lin, 2019^12^	R	796	OPSCC HPV+	CT‐MRI‐PET/CT	LN short axis >5 mm, long axis ≥ 10 mm, presence of any medial RLN; central necrosis; ≥2 clustered RPLNs; SUV >4.5	RT/RTCT
Bhattasali, 2018^19^	R	238	cT1‐2 N1 OPSCC HPV +	CT‐MRI‐PET/CT	N/A	RT/ CTRT
Park, 2019^32^	R	71	Tonsil cancer	CT‐MRI‐PET/CT	N/A	S
Troob, 2017^33^	R	30	OPSCC	CT‐PET/CT	N/A	S
Spector, 2016^20^	R	205	Stage III/IV OPSCC	CT‐PET/CT	LN > 10 mm, abnormal SUV, cystic or necrotic, rENE	RT/RTCT
Baxter, 2015^29^	R	165	OPSCCs HPV+	CT‐MRI‐PET/CT	LN abnormal SUV, LN short axsis >6 mm, central necrosis or clustered.	RT/ CTRT
Chung 2015^36^	R	54	OPSCC	CT‐MRI‐PET/CT	N/A	S
Samuels, 2015^30^	R	231	OPSCC HPV +	CT‐MRI‐PET/CT	LN long axis >1 cm, necrotic/ cystic, abnormal SUV.	RT/RTCT
Gunn, 2013^8^	R	981	OPSCC	CT‐MRI‐PET/CT	LN short axis >5 mm or long axis > 10 mm; necrosis, hypodensity; >1 lateral RLN, SUV >4,5; any medial RLN.	RT/RTCT
Moore, 2013^35^	R	72	OPSCC	CT‐MRI‐PET/CT	N/A	S
Tang, 2013^10^	R	164	OPSCC	CT‐MRI‐PET/CT	SUV > 3 or LN short axis >6 mm.	RT/RTCT
Chung, 2011^34^	R	76	Tonsil cancer	CT‐MRI‐PET/CT	N/A	S
Chan, 2010^22^	P	102	OPSCC	PET/CT	Any medial RLN, abonormal SUV, LN short axis >5 mm	RT/ CTRT
Tauzin, 2010^31^	R	53	OPSCC	PET/CT	SUV >3, LN ≥10 mm, any suspicious feature.	RT/RTCT
Chu, 2009^37^	R	29	OSCC, OPSCC, HPSCC	CT‐MRI‐PET/CT	LN >8 mm, abnormal density/ asymmetry/ enhancement. SUV >2,5	S
Yoshimoto, 2007^21^	P	84	OPSCC	Histopathology	N/A	S OR RT/RTCT
Dirix, 2006^7^	R	208	OPSCC	CT	LN axis > 10 mm or central hypodensity	S OR RT/RTCT
Shimizu, 2006^9^	R	77	OPSCC	Histopathology	N/A	S
McLaughlin, 1995^23^	R	443	H&N	CT‐MRI	LN > 10 mm, central hypodensity.	S OR RT/RTCT

Abbreviations: LN, lymph node; P, Prospective; PET, Positron emission tomography; R, Retrospective; rENE, radiological extra nodal extension; RLN, Retropharyngeal lymph node; SUV, standardized uptakevalue; OPSCC, oropharyngeal squamous cell carcinoma; OSCC, oral squamous cell carcinoma; HPSCC, hypopharyngeal squamous cell carcinoma; H&N, head and neck.

### 3.2 Quality assessment

The quality of included studies was high (Newcastle‐Ottawa Scale score ≥7) in 18 (81%) of 22 studies, with a median of 7 (interquartile range: 7–8). A detailed report on the quality of included studies according to the Newcastle‐Ottawa Scale is reported in Supplementary Table 1.

### 3.3 Prevalence of metastatic RLNs in OPSCC

**FIGURE 2:**
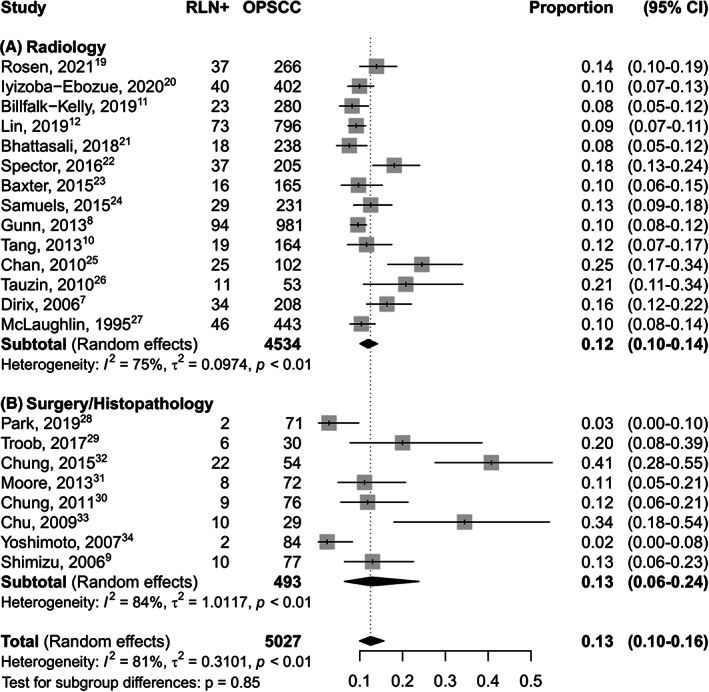
Forest plot showing the pooled prevalence of RLN metastasis in OSCC

All the eligible studies provided information about the prevalence of the metastatic involvement of RLN in OPSCC. When more subsites of the head and neck were analyzed, only data about OPSCCs were extracted. As shown in Figure 2, the pooled prevalence of RLN in OPSCC was 0.13 (CI 95% 0.10–0.16). The pooled prevalence of RLNMs was 0.13 (CI 95% 0.06–0.24) and 0.12 (CI 95% 0.10–0.14) when evaluated by histopathological analysis and imaging findings, respectively. Table 1 summarizes all criteria adopted by authors for this purpose. Among dimensional criteria, three authors^11^, ^27^, ^37^ used an 8 mm cut‐off, seven^7^, ^8^, ^12^, ^20^, ^23^, ^30^, ^31^ 10 mm, and four a short axis >5 mm. As qualitative or metabolic features, central necrosis or hypodensity, clustered LN,^8^, ^12^, ^20^, ^28^, ^29^, ^30^, ^31^, ^37^ medial location, and Standardized Uptake Value (SUV) >4.5^8^, ^12^ have been generally considered as pathologic. No medial RLNMs were reported in any of the seven studies providing data about their involvement.

### 3.4 Prevalence of metastatic RLN according to HPV status

**FIGURE 3:**
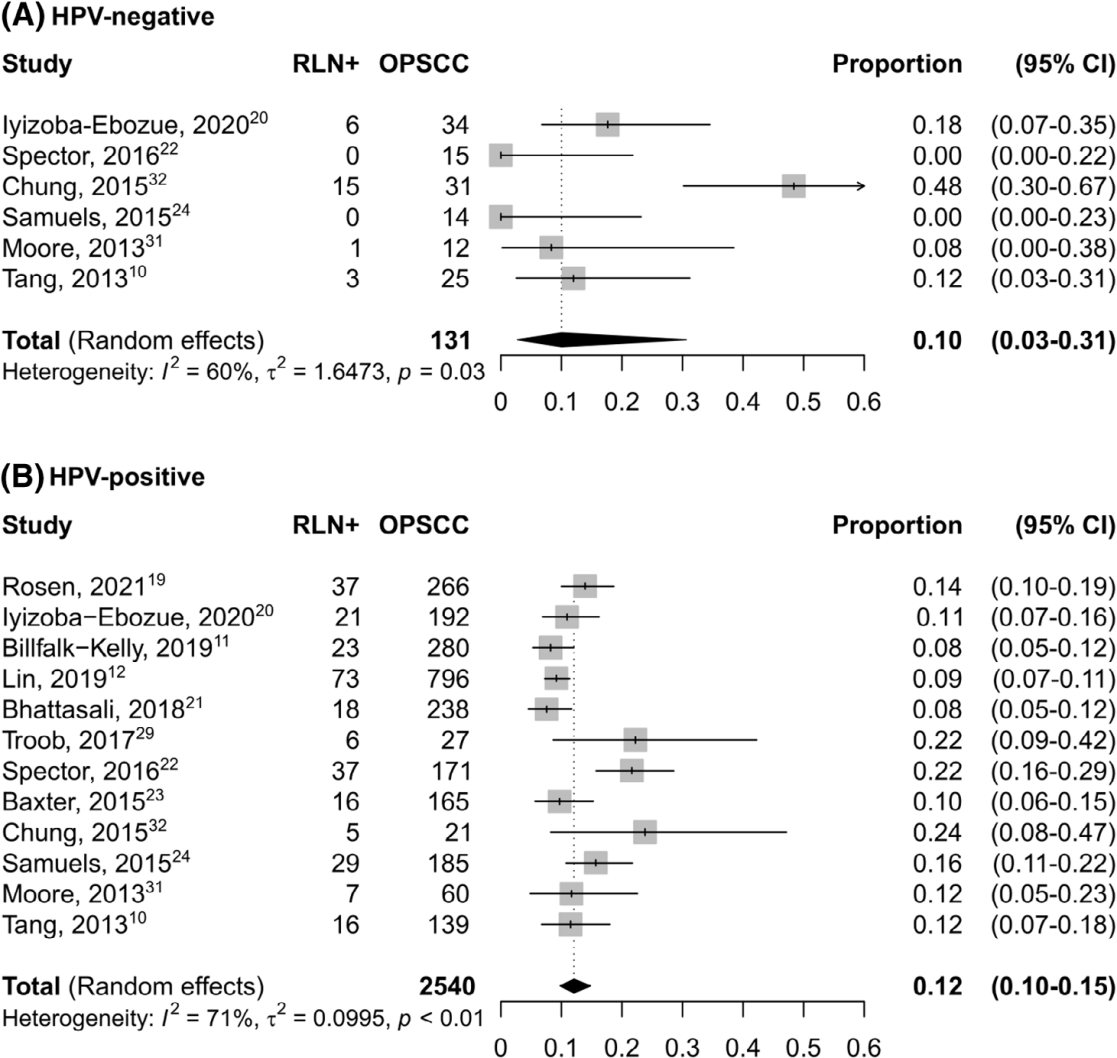
Forest plot showing the pooled prevalence of RLN metastasis in OSCC according to HPV‐status

When stratified according to HPV status (Figure 3), the prevalence of RLN metastasis was 0.10 (95% CI 0.03–0.31) in HPV‐negative cancers, while it was 0.12 (95% CI 0.10–0.15) in HPV‐positive ones.

### 3.5 Prevalence of metastatic RLN according to oropharyngeal subsites

**FIGURE 4:**
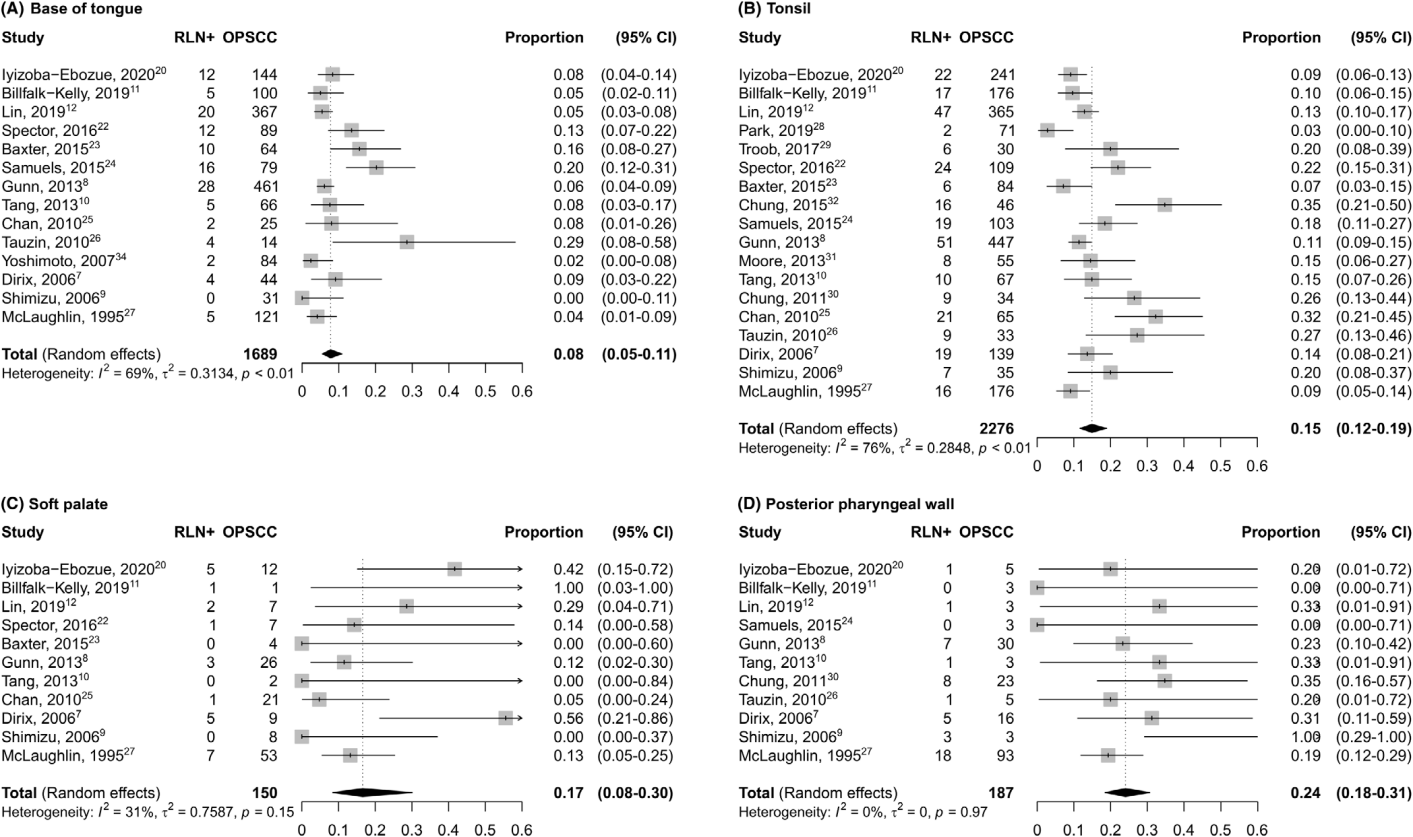
Forest plot showing pooled prevalence of RLN metastasis for different oropharyngeal subsites (A) Base of tongue (B) Tonsil (C) Soft palate (D) Posterior pharyngeal wall

Oropharyngeal subsites were divided into tonsil, base of tongue and anterior pharyngeal wall, posterior and lateral pharyngeal wall, and soft palate or superior pharyngeal wall. Three studies did not provide a specific oropharyngeal localization.^19^, ^27^, ^37^ Figure 4 shows the prevalence of RLN metastasis according to oropharyngeal subsite. Posterior and lateral pharyngeal wall was the subsite with the highest prevalence of RLN metastasis (Figure 4D) with 0.24 (95% CI: 0.18–0.31), followed by soft palate (Figure 4C), 0.17 (0.08–0.30). The prevalence of RLN metastasis was 0.15 (95% CI: 0.12–0.19) in tonsillar cancer (Figure 4B), while base of tongue (Figure 4A) showed the lowest frequency of involvement 0.08 (95%CI 95% CI: 0.05–0.10).

### 3.6 Outcome measures

**FIGURE 5:**
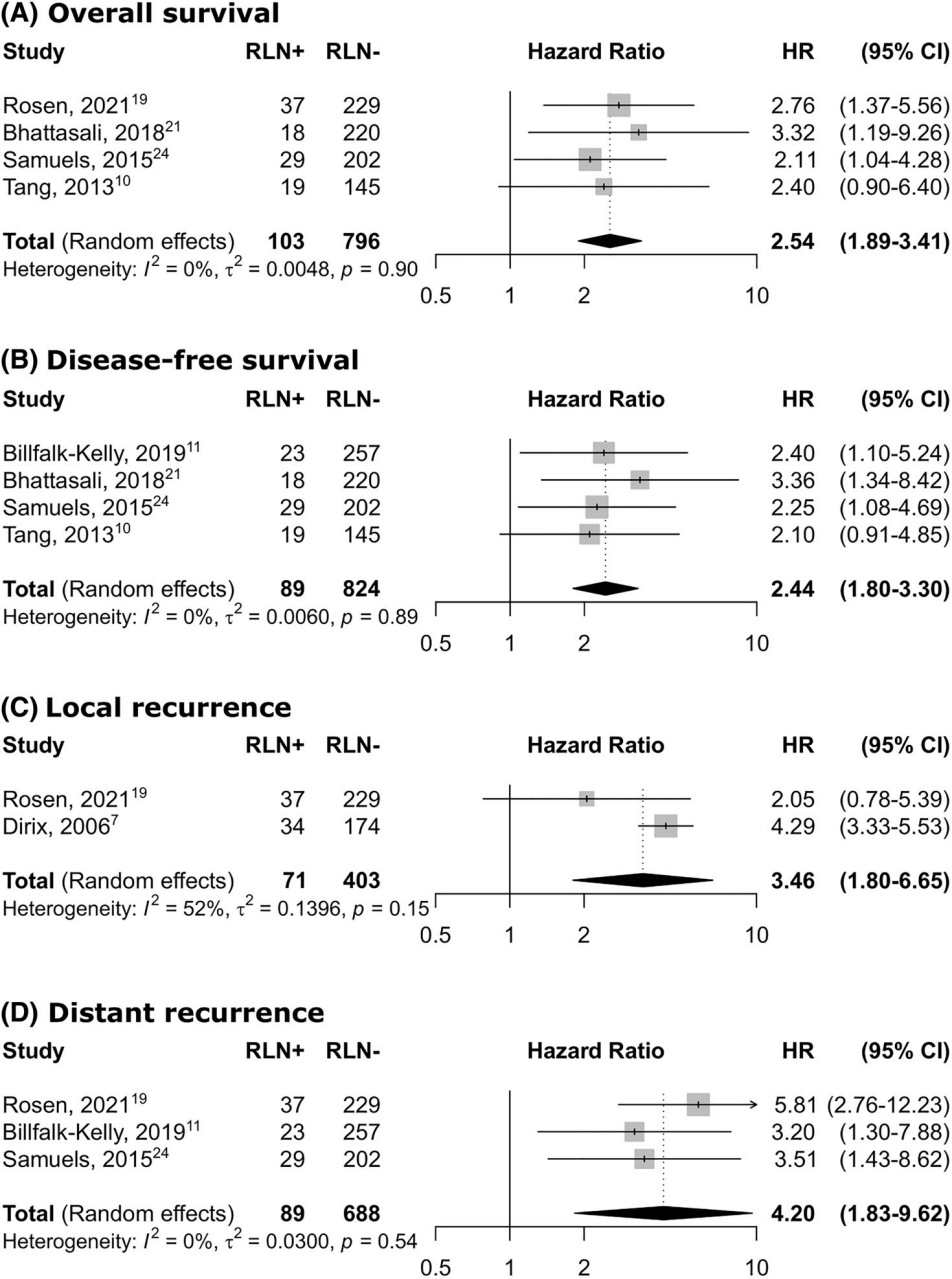
Forest plot showing hazard ratios for overall survival, disease free survival, locoregional recurrence and distant recurrence

Six articles were eligible for survival analysis. Among them only one^10^ did not perform multivariate analysis. All others^7^, ^11^, ^19^, ^27^, ^30^ specified which parameters were included in multivariate analysis. T category, N category, age, and smoking status were included in all studies which performed multivariate analysis. Four articles provided HRs and 95% CIs for OS.^10^, ^19^, ^27^, ^30^ As shown in Figure 5, patients with metastatic involvement of the RLN had a significantly poorer OS than those without RLNMs (sHR  =  2.54, 95% CI: 1.89–3.41). There was no significant heterogeneity among these four articles (*I* ^2^  =  0%, *p*  = 0.90). Four articles provided HRs and 95% CIs of DFS,^10^, ^11^, ^19^, ^30^ revealing that patients with metastatic involvement of the RLN had a significantly poorer DFS than those without RLNMs (HR  =  2.44, 95% CI: 1.80–3.30). No significant heterogeneity was observed (*I* ^2^  =  0%, *p*  = 0 .89). Only two articles^7^, ^27^ provided HRs and 95% CIs of LRC: RLN positive patients were at higher risk for loco(regional) recurrence (HR  =  3.46, 95% CI: 1.80–6.65). Finally, concerning DM, three studies^11^, ^27^, ^30^ were included: RLN positive patients were also at higher risk for distant metastases (HR = 4.20, 95% CI: 1.83–9.62).

### 3.7 Publication bias and sensitivity analysis

We inspected publication bias through a funnel plot. The result (Supplementary Figure 1A) indicated a lack of publication bias (test for asymmetry in funnel plot: *p* = 0.295). Furthermore, influence analysis was conducted on the estimation of prevalence of RLN metastases: no relevant impact emerged by any study (Supplementary Figure 1B), with prevalence estimates ranging from 12% to 13%.

## 4 DISCUSSION

Combining data from 22 studies, the pooled prevalence of RLNMs in OPSCC was 13.0%. No significant differences emerged according to HPV status and the method used to assess RLN metastatic involvement. On the other hand, RLNMs were observed to be more prevalent in cancers of the posterior pharyngeal wall compared with other subsites. Finally, RLNMs were associated with an unfavorable prognostic outcome in patients with OPSCC, doubling the risk of death and progression and increasing by more than 3 times the risk of local and distant recurrence.

According to our pooled data, several individual studies were consistent in reporting a higher prevalence of RLNMs in patients with SCC of the posterior pharyngeal wall.^18^, ^28^ Chung et al. suggested that this may be due to the contiguity of the retropharyngeal space with the pharyngeal wall.^34^, ^36^ On the contrary, tonsil and BOT, which account for the vast majority of OPSCC cases,^20^ were less associated with RLNMs. The tonsil and BOT drain first to jugulodigastric nodes, and only then to the retropharyngeal space^2^, ^24^, ^43^ with only the involvement of the posterior pillar of the tonsil increasing the risk of RLNMs.^2^, ^20^ The incidence of OPSCC is increasing worldwide, due to the emerging role of high risk strains of HPV in oropharyngeal carcinogenesis.^44^ The attributable fraction of HPV‐driven OPSCC is globally 31% and it is higher for SCC arising from the palatine tonsil and BOT^45^ as opposed to SCC arising from the soft palate and posterior pharyngeal wall, which are very rarely caused by HPV infection but are also less frequent (4.4% in the present systematic review).^46^ Thus, as the majority of HPV‐negative OPSCC still arise from the tonsil and BOT, an expected at least indirect association between HPV‐status and the risk of RLNMs was not observed in the present meta‐analysis.

Among the seven eligible papers that reported information about medial RLN,^8^, ^10^, ^12^, ^21^, ^28^, ^30^, ^32^ none found medial RLN involvement. Bussels et al. found only one patient out of 208 (0.5%) with medial RLNMs.^18^ This patient also had a pathologic contralateral lateral RLN and the primary tumor was located in the posterior pharyngeal wall. Kim et al. found five medial RLNMs (11.6%) in a cohort of HNSCC where oropharyngeal and hypopharyngeal cancers counted for 54% and 39% of patients, respectively.^42^ Medial RLN are usually not evident in adults, therefore, their presence must be considered pathognomonic for metastatic involvement.^6^, ^29^, ^47^, ^48^ Medial RLNMs are also less common than involvement of the lateral RLN in NPC, with one report identifying them only in six patients out of 3100 (0.2%).^49^ Unfortunately, no studies have been conducted specifically to investigate their clinical impact and prevalence in OPSCC; rather they have been reported only as collateral findings.^6^, ^29^, ^47^, ^48^

Only six articles reported sufficient survival data and could therefore be included in this meta‐analysis. Results demonstrate a statistically significant trend towards unfavorable prognosis in patients with RLNMs in terms of OS, DFS and recurrence.^7^, ^10^, ^11^, ^19^, ^27^, ^30^ However, there is a lack of consensus in the literature on the topic. Indeed, several studies reported no statistical differences in terms of outcome.^10^, ^12^, ^29^ All of these had in common the fact that their cohorts were HPV positive. In the study by Tang et al.^10^ despite multivariate analysis not having been performed, OS and event‐free survival became non‐significant when only the HPV positive population was considered. On the other hand, in studies that did not consider HPV status,^8^, ^34^ survival outcomes trended towards significantly worse outcomes even at multivariate analysis.

However, differences in reported rates of RLNMs may not be attributable to HPV status alone. As hypothesized by Gross et al.,^50^ appropriate multimodal therapy may provide survival benefits overcoming any possible negative influence deriving from RLNMs. It is possible that since RLNMs are associated with higher N and T grade it may simply be a marker of advanced disease,^10^, ^18^ although when multivariate analysis was performed, as in almost all the reports included in our metanalysis, the effect of T and N category on prognosis faded, leaving RLNM as an independent prognostic factor towards worse outcomes. All of these factors may explain why there is still no sharp definition of the effect of RLNMs on prognosis.

This systematic review also highlights the difficulty in diagnosing RLNMs. CT alone is not sufficiently accurate,^4^ although the introduction of PET/CT has given rise to a powerful diagnostic tool based on multimodal imaging using CT, PET/CT and MRI. Indeed, PET/CT has been shown to increase the accuracy in diagnosing RLNMs to 86.7% from 60.6% in those imaged with CT and/or MRI, with similar improvements in sensitivity, specificity, positive predictive value, and negative predictive value.^37^ Meta‐analysis of sensitivity, specificity, and accuracy of imaging studies has not been possible since different studies used different diagnostic tools, moreover different criteria have been used to address RLN as metastatic. Most investigators used a multimodality imaging strategy, which was proven to have a high accuracy in detecting RLN metastasis.^7^, ^11^, ^23^ This is consistent with the similar prevalence of RLNMs observed in imaging (12%) and histopathological studies (13%).

Some study limitations have to be acknowledged. Firstly, the included studies used a heterogeneous TNM classification, in fact, the time span of the eligible articles vary from AJCC 2nd ed. to AJCC 8th ed. Moreover, clinical records are not uniform across centers in terms of treatment and diagnostic criteria. RLN specimens for histologic examination are difficult to obtain, thus diagnosis relies primarily upon radiological assessment, with various criteria and modalities used by different centers. Reporting of N and T categories were only slightly heterogeneous, being included in the multivariate analysis in five out of six articles. In addition, the inclusion of previous studies was limited by the frequent lack of data required for the meta‐analysis: indeed, a number of studies displayed Kaplan–Meyer curves without reporting hazard evaluations, thus considerably diminishing the amount of data available. Finally, few studies reported data regarding RLNMs according to primary tumor subsite, which limited this analysis.

**FIGURE 6:**
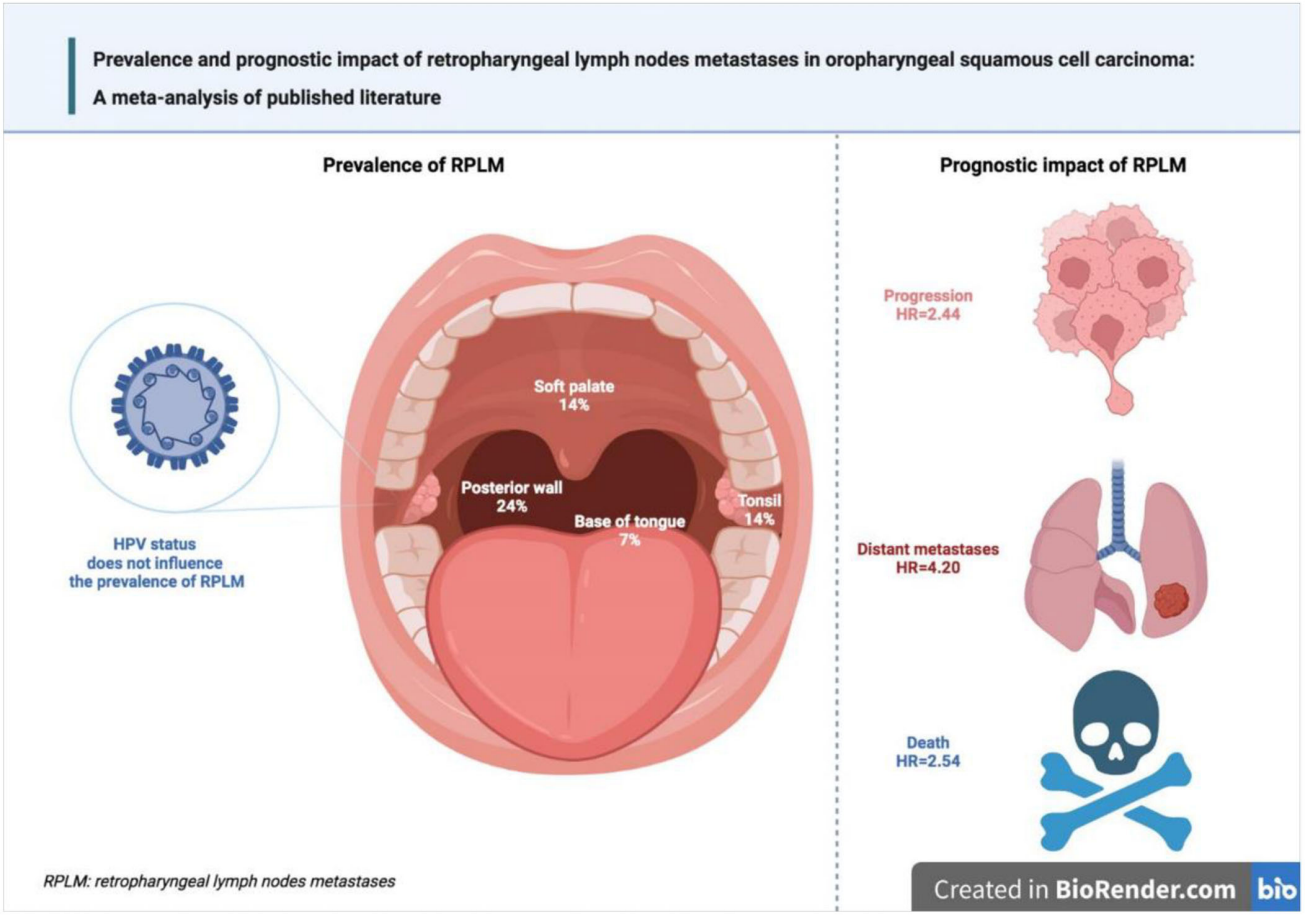
Main results emerged from the meta‐analysis [Color figure can be viewed at wileyonlinelibrary.com]

In conclusion (Figure 6), this meta‐analysis found that one in 10 OPSCC patients harbored RLNMs, with cancers arising from the posterior pharyngeal wall having the highest prevalence. RLNMs were associated with unfavorable prognostic outcomes. However, these results must be considered with caution since, given the paucity of data available in the literature, this meta‐analysis was not based only on individual studies fully adjusted for possible confounding factors.

## AUTHOR CONTRIBUTIONS

Giancarlo Tirelli, Nicoletta Gardenal, Enrico Zanelli, Daniele Borsetto, Veronica Philipps, Alberto Vito Marcuzzo, Jonathan Fussey, Jerry Polesel, and Paolo Boscolo‐Rizzo contributed equally in conception, design, analysis, and interpretation of data.

## CONFLICT OF INTEREST

The authors declare no conflict of interest.

## Supporting information


**APPENDIX S1** Supporting information

## Data Availability

The data that support the findings of this study are available from the corresponding author upon reasonable request.
