# Supplementary material for: Prevalence and prognostic impact of retropharyngeal lymph nodes metastases in oropharyngeal squamous cell carcinoma: Meta‐analysis of published literature
Source: Head Neck. 2022 Aug 1;44(10):2265–76. doi: 10.1002/hed.27166 (PMC9540534; doi:10.1002/hed.27166)

**(Supplementary material) Table S1- Quality assessment**

|  | **Representativeness of the exposed cohort** | **Selection of the non-exposed cohort** | **Ascertainment of exposure** | **Demonstration that outcome of interest was not present at start of study** | **Comparability of cohorts on the basis of the design or analysis controlled for confounders **** | **Assessment of outcome** | **Was follow-up long enough for outcomes to occur** | **Adequacy of follow up of cohorts** | **Total score**  **[0-9]** |
| --- | --- | --- | --- | --- | --- | --- | --- | --- | --- |
| **Rosen BS, 2021** | * | * | * | * | * | * | * | * | 8 |
| **Billfalk, 2019** | * | * | * | * | * | * | * | * | 8 |
| **Lin, 2019** | * | * | * | * | * | * | * | * | 8 |
| **Iyizoba-Ebozue, 2020** | * | * | * | * | ** | * | * | * | 9 |
| **Bhattasali, 2019** | * | / | * | * | * | * | * | * | 7 |
| **Park, 2018** | * | / | * | * | * | * | * | * | 7 |
| **Troob, 2017** | * | * | * | * | * | * | * | * | 8 |
| **Spector, 2016** | * | / | * | * | * | * | * | * | 7 |
| **Baxter, 2015** | * | * | * | * | / | * | * | * | 7 |
| **Samuels, 2015** | * | * | * | * | ** | * | * | * | 9 |
| **Chung, 2015** | * | * | * | * | * | * | * | * | 8 |
| **Gunn, 2013** | * | * | * | * | * | * | * | * | 8 |
| **Moore, 2013** | * | * | * | * | ** | * | * | * | 9 |
| **Tang, 2013** | * | * | * | * | / | * | * | * | 7 |
| **Chung, 2011** | * | / | * | * | * | * | * | * | 7 |
| **Tauzin, 2010** | * | / | * | * | / | * | * | * | 6 |
| **Chan, 2010** | * | / | * | / | * | * | * | * | 6 |
| **Chu, 2009** | * | * | * | * | / | * | * | * | 7 |
| **Yoshimoto, 2007** | * | * | * | * | / | * | * | * | 7 |
| **Dirix, 2006** | * | * | * | * | * | * | * | * | 8 |
| **Shimizu, 2006** | * | / | * | * | / | * | * | * | 6 |
| **McLaughlin, 1995** | * | / | * | * | * | * | * | * | 7 |

**(Supplementary material) Figure S1- (A) Funnel Plot for publication BIAS; (B) Influence analysis.**
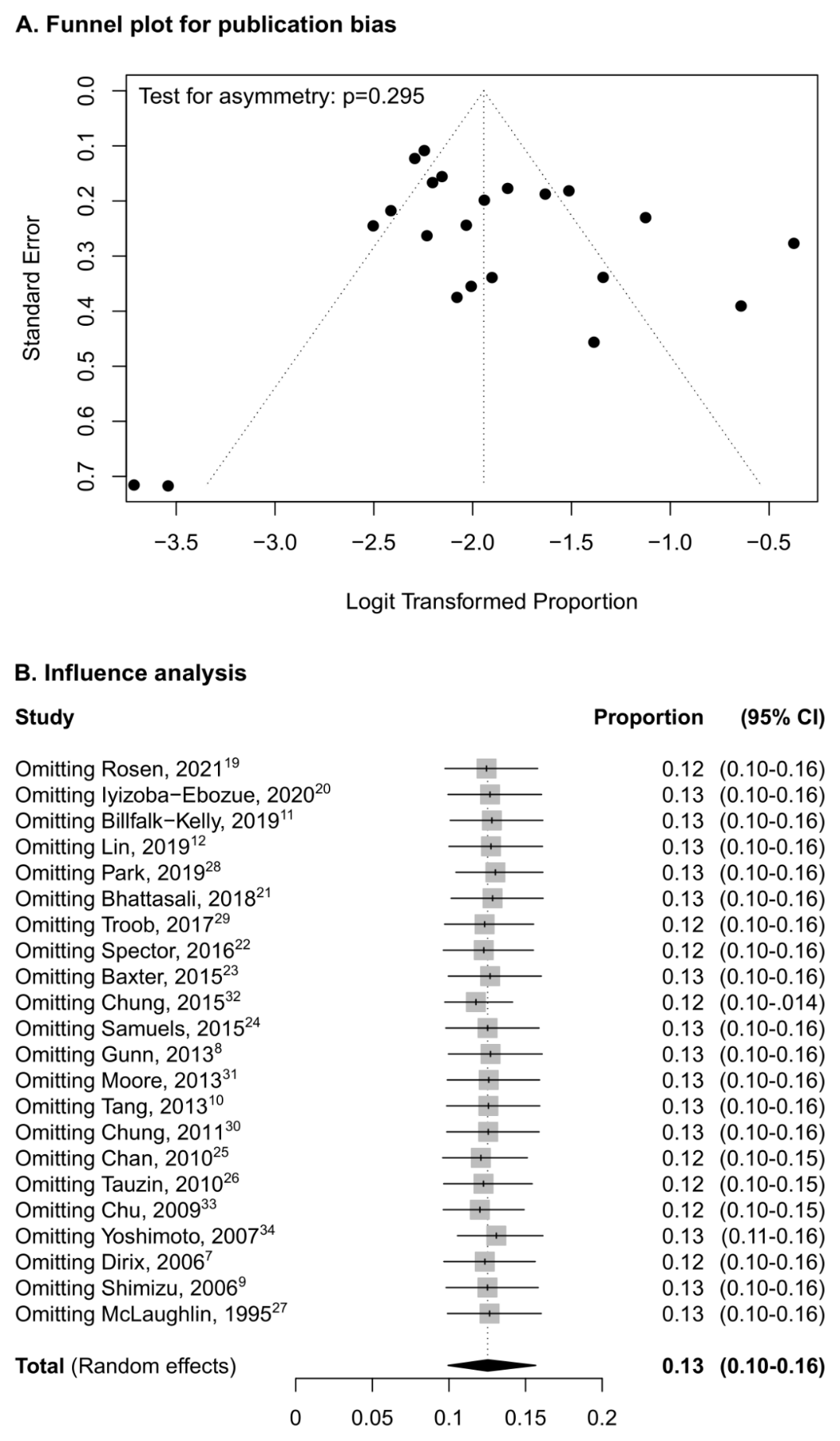

Supplement: Supplementary file 1 — APPENDIX S1 Supporting information [file HED-44-2265-s001.docx]
